# Supplementary material for: Study of the epidemiological behavior of malaria in the Darien Region, Panama. 2015–2017
Source: PLoS One. 2019 Nov 15;14(11):e0224508. doi: 10.1371/journal.pone.0224508 (PMC6857920; doi:10.1371/journal.pone.0224508)
Supplement: Attachment — Submitted filename: Response to reviewers.doc [file pone.0224508.s001.doc]

Panama, July 8, 2019

Doctor

***Luzia Helena Carvalho, PhD.***

Academic Editor PLOS ONE

1160 Battery Street

Koshland Building East, Suite 225

San Francisco, CA 94111, USA

Dear Editor

Please find below the corrections made to the manuscript PONE-D-19-14773 “**Study of the epidemiological behavior of malaria in the Darién Region, Panama. 2015 - 2017. Epidemiology of malaria in Darién”,** following the recommendations and observations of the reviewers and editors of PLOS ONE.

**Initial editorial review:**

1. The manuscript was corrected according to the style requirements of PLOS ONE.

2. The declaration of ethics was modified according to the recommendations and style of PLOS ONE.

3. The way the informed consent was obtained was explained.

4. The following references were corrected:

# [**https://doi.org/10.1186/s12936-018-2235-3**](https://doi.org/10.1186/s12936-018-2235-3) (Climatic fluctuations and malaria transmission dynamics, prior to elimination, in Guna Yala, República de Panamá).

## [***https://doi.org/10.1155/2018/3954717***](https://doi.org/10.1155/2018/3954717) (Evaluating Malaria Prevalence Using Clinical Diagnosis Compared with Microscopy and Rapid Diagnostic Tests in a Tertiary Healthcare Facility in Rivers State, Nigeria).

# [**https://doi.org/10.1038/s41598-018-23801-9**](https://doi.org/10.1038/s41598-018-23801-9) (Micro-epidemiology of mixed-species malaria infections in a rural population living in the Colombian Amazon region).

## [***https://doi.org/10.1155/2018/9163543***](https://doi.org/10.1155/2018/9163543) (Insecticide Resistance and Its Intensity in Populations of Malaria Vectors in Colombia).

5. Figures 1,2,4 and 6, and all maps were prepared by the Geographic Information Department of ICGES.

6. Figure 7 was eliminated due to the short time to achieve the signature of the participants and to be able to send the manuscript on the date indicated by PLOS ONE.

**Reviewer 1.**

**Major Comments**

**1. Objective and presentation of results**

• The objective was corrected and modify according to the results of the study.

• The results indicated that the intervention measures used by NMP were the use of IRS, application of insecticide through the use of thermospray in places where outbreaks or cases of malaria were registered, application in anopheline breeding grounds of *Bacillus sphaericus* (Vectolex®) and the active and passive search for suspicious cases.

• The evaluation was carried out taking into consideration the regional malaria control plan, intersectoral cooperation, coverage of interventions, access to malaria diagnosis and treatment and the epidemiological surveillance system.

**2. Materials and methods**

• The description of the study design was corrected as follows: A retrospective analysis was carried out to determine the incidence, prevalence and distribution of malaria in the Darién region, considering all cases diagnosed by active and passive search by the PNM during the 2015-2017 period.

• In the paragraph that indicates "Malaria is a disease of mandatory notification in Panama", this includes all cases of malaria detected by active search by the NMP and passive by health facilities.

**3. Tables 4,5 and 6**.

The confidence intervals in tables 4,5 and 6 were eliminated considering the suggestion of Reviewer 2.

**Minor Comments**

**1. Summary**

- Information on the age group and sex with malaria, the sentence was corrected as follows: 62.7% (n = 314) of the cases occurred in males and 65.5% (n = 328) of the total cases occurred in economically active age groups.

**2. Introduction: paragraph 1-4**

• The words autochthonous and Mesoamerica / anthropogenic were defined

• The National Malaria Program (NMP) was placed in parentheses when it was first written.

• Point (.) Was removed on line 3 of paragraph 4.

• The word ethnicity was deleted in order to make it clear that all human groups are ethnic groups.

**3.** API, SPR and ABER were defined. It was specified how each of these indicators were calculated.

**4. Results: Malaria indicators**

• In reference to the age group of 1 to 59 years that had a higher incidence of malaria, the sentence was corrected, as follows: People between the ages of 1 to 59 years of age presented a higher malaria incidence (n = 455 ; 91.0%) compared to the rest of the age groups.

**5. Results: Migration and malaria**

• Regarding observations on gametocytes, in diagnostic and treatment methods, we added that the number of samples with the presence of gametocytes was considered for analysis due to their epidemiological importance. In the results of diagnosis and treatment, we also added that 43.5% (n = 218) of the diagnosed cases showed the presence of gametocytes, and in Table 4, the number of cases with the presence of gametocytes was added.

**6. Table 2**

• A table was created for the image capture of Table 2, This Table was divided into two tables (Table 1 and Table 1a).

**7. Table 4 (now Table 3)**

In Table 3; NI = There is no information about the age group. The asterisk (*) was removed.

**b**. which represents the column.

In the column the title was corrected and “Total cases by age” was placed, where reference is made to the total number of cases by age group.

**8. Results: signs and symptoms (and table 6)**

It is important to indicate that although the signs and symptoms of malaria are known in general, they can vary and present a broad clinical spectrum that can result in asymptomatic infection, uncomplicated and fatal malaria according to the species of the parasite and its genotype, the human host and the environment. Similarly, people who live in endemic sites and especially adults get to develop some immunity compared to people who live in non-malarial sites where the disease can manifest itself in the population in a more severe way. For these reasons, it was considered to study and analyze the main signs and symptoms of the disease in people diagnosed with malaria. This type of analysis has not been done in this region of the country; thus we consider it important.

**9. Table 6**

The confidence interval columns in the table were eliminated following recommendation of Reviewer 2.

**Reviewer 2.**

**•** The manuscript was edited, and several paragraphs were deleted to shorten it.

**•** In summary, the objective was corrected as follows: The objective of the study was to determine the main characteristics of the epidemiological behavior of malaria in the Darién region.

**•** Figure 2 and Table 1 were removed considering the reviewer's suggestion.

**•** The population size was added in Table 2.

**•** In Tables 3,4 and 5, the columns with the confidence intervals were eliminated.

**•** The data in Table 3 was only used to analyze the number of cases by age group and sex.

**•**Table 5, about signs and symptoms of malaria: Although the signs and symptoms of the disease are generally known, however, as indicated above, they can vary and present a broad clinical spectrum that can result in asymptomatic infection, uncomplicated and lethal malaria according to the species of the parasite and its genotype, the human host and the environment. Similarly, people who live in endemic sites and especially adults can develop some immunity compared to people who live in non-malarial sites where the disease can manifest itself in the population in a more severe way. For these reasons, it was determined to know and analyze the main signs and symptoms of the disease in people diagnosed with malaria. This type of analysis has not been done in this region of the country, so we consider it important to analyze it to see if with subsequent studies that are done you can find differences.

**•** The discussion was shortened. Aspects related to migration, antimalarial treatment and control measures were modified according to the results.
