# Supplementary material for: Study of the epidemiological behavior of malaria in the Darien Region, Panama. 2015–2017
Source: PLoS One. 2019 Nov 15;14(11):e0224508. doi: 10.1371/journal.pone.0224508 (PMC6857920; doi:10.1371/journal.pone.0224508)
Supplement: Attachment — Submitted filename: Response to Reviewers - October 2019.doc [file pone.0224508.s002.doc]

Panama, October 8, 2019

Doctor

***Luzia Helena Carvalho, PhD.***

Academic Editor PLOS ONE

1160 Battery Street

Koshland Building East, Suite 225

San Francisco, CA 94111, USA

Dear Editor

Please find below the corrections made to the manuscript PONE-D-19-14773 “**Study of the epidemiological behavior of malaria in the Darién Region, Panama. 2015 - 2017. Epidemiology of malaria in Darién”,** following the recommendations and observations of the reviewers and editors of PLOS ONE.

**Reviewer #1:**

As suggested, the word “prevalence” was eliminated and replace by the word “incidence” throughout the manuscript.

**Reviewer #2:**

Referring to Table 1., the word Table 1a was deleted and added below.

In Table 2, the definitions of the API, SPR and ABER malariometric indicators were added.

In Table 4, the “No. of gametocyte” column was moved to the right side.

In Table 5, a column with the “signs and symptoms” for *P. falciparum* was added and separated from *P. vivax*. Also a comparison was made.
